# Major Adverse Cardiac Events After Gastric Bypass vs Sleeve Gastrectomy

**DOI:** 10.1001/jamasurg.2025.1065

**Published:** 2025-05-07

**Authors:** Simone Wildisen, Rahel Laager, Tristan Struja, Alessia Wildisen, Beat Mueller, Philipp Schuetz, Ralph Peterli, Alexander Kutz

**Affiliations:** 1 Medical University Clinic, Kantonsspital Aarau, Aarau, Switzerland; 2 University Hospital of Child and Adolescent Psychiatry and Psychotherapy, University of Bern, Bern, Switzerland; 3 Department of Clinical Research, Medical Faculty of the University of Basel, Basel, Switzerland; 4 Institute for Medical Engineering and Science, Massachusetts Institute of Technology, Cambridge, Massachusetts; 5 Department of Adult Psychiatry and Psychotherapy, University Hospital of Psychiatry Zurich, Zurich, Switzerland; 6 Medical University Clinic, Division of Endocrinology, Diabetes and Metabolism, Kantonsspital Aarau, Aarau, Switzerland; 7 Clarunis, University Digestive Health Care Center, St Clara Hospital and University Hospital Basel, Basel, Switzerland; 8 Division of Pharmacoepidemiology and Pharmacoeconomics, Department of Medicine, Brigham and Women’s Hospital and Harvard Medical School, Boston, Massachusetts

## Abstract

**Importance:**

Metabolic bariatric surgery is the most effective and durable treatment for weight loss and improvement of cardiovascular diseases. With sleeve gastrectomy now surpassing gastric bypass as the most common procedure worldwide, comparing these procedures’ associations with major adverse cardiac events (MACE) is needed.

**Objective:**

To compare the risk of MACE among individuals undergoing gastric bypass or sleeve gastrectomy.

**Design, Setting, and Participants:**

This population-based, inverse probability–weighted cohort study was conducted using administrative claims data from Switzerland among adults undergoing gastric bypass or sleeve gastrectomy for obesity treatment between January 2012 and December 2022. Inpatient individuals with a primary or secondary discharge procedure code for gastric bypass or sleeve gastrectomy were eligible for inclusion. Data were analyzed from April 2024 to September 2025.

**Exposure:**

Gastric bypass vs sleeve gastrectomy.

**Main Outcomes and Measures:**

A weighted cohort was analyzed to study the primary outcome of 4-point MACE, including acute myocardial infarction, ischemic stroke, hospitalizations for heart failure, and all-cause mortality. Secondary outcomes were the individual components of MACE, surgical reinterventions, and associated complications.

**Results:**

Of 39 067 patients, 30 270 patients (77.5%) underwent gastric bypass and 8798 patients (22.5%) underwent sleeve gastrectomy. Median (IQR) patient age was 42 (35-50) years, and 28 560 patients (73.1%) were women. A total of 23 708 patients (60.7%) had a body mass index (calculated as weight in kilograms divided by height in meters squared) of 40 or higher. After weighting, over a median (IQR) follow-up of 5.1 years (2.6-7.6), the primary outcome occurred in 577 patients in the gastric bypass group (1.9%) and 264 patients in the sleeve gastrectomy group (3.0%), with incidence rates of 3.96 and 5.10 per 1000 patient-years, respectively (hazard ratio [HR], 0.75; 95% CI, 0.64-0.88). This difference was primarily driven by lower rates of acute myocardial infarction (HR, 0.63; 95% CI, 0.46-0.86). No differences were observed in ischemic stroke, hospitalization for heart failure, and all-cause mortality. Both short- and long-term secondary outcomes favored gastric bypass over sleeve gastrectomy, except for higher rates of revision surgery and immediate postoperative complications.

**Conclusions and relevance:**

In this inverse probability–weighted cohort study, for patients undergoing metabolic bariatric surgery, gastric bypass was associated with lower rates of MACE than sleeve gastrectomy over a follow-up period of up to 11 years. Known postoperative complications were confirmed for both gastric bypass and sleeve gastrectomy.

## Key Points

**Question:**

Is there a difference in major adverse cardiac events (MACE) between patients with obesity undergoing gastric bypass vs sleeve gastrectomy?

**Findings:**

In this population-based cohort study of 39 067 adults with up to 11 years of follow-up, gastric bypass was associated with a lower risk of MACE compared with sleeve gastrectomy, primarily due to reduced rates of myocardial infarction. Safety outcomes were consistent with previous clinical trials.

**Meaning:**

Patients undergoing gastric bypass had a lower risk of MACE compared to those undergoing sleeve gastrectomy, suggesting a cardiovascular advantage of gastric bypass over sleeve gastrectomy.

## Teaser

This cohort study conducted in Switzerland compares the risk of major adverse cardiac events among individuals undergoing gastric bypass or sleeve gastrectomy for obesity treatment.

## Introduction

Obesity and its associated comorbidities are an increasing global health burden, with prevalence reaching unprecedented levels. In 2022, 1 in 8 individuals worldwide was living with obesity.^1,2^ Recent estimates indicate that nearly half of all adults in the US will be obese in 2030.^3^ In Switzerland, the prevalence of obesity has already doubled to approximately 12% in 2022, with a continuing upward trend.^4^

The relationship between obesity, morbidity, and mortality is well established, as is the metabolic benefit of weight loss in managing obesity-related comorbidities, such as type 2 diabetes, hypertension, hyperlipidemia, and obstructive sleep apnea—all of which are risk factors for atherosclerotic cardiovascular disease.^5,6,7,8^

New obesity management medications, such as glucagon-like peptide-1 receptor agonists, have demonstrated promising results for weight reduction; however, sustainable long-term weight loss, especially after cessation of the drugs, has yet to be confirmed.^9,10^ Additionally, challenges persist with medication adherence and availability.^11^ Until further research provides more definitive long-term outcomes, metabolic bariatric surgery (MBS) remains the most effective and sustainable treatment for severe obesity.^12,13^

Currently, sleeve gastrectomy (SG) is the most frequently performed bariatric procedure worldwide, followed by gastric bypass (GB); SG has gained attraction due to its faster, simpler procedure and excellent outcomes comparable to GB.^14,15^ Although GB is a more complex procedure involving the small bowel, it remains the criterion standard in Switzerland for achieving weight loss and type 2 diabetes remission.^14,16,17^ GB is reversible and less prone to suboptimal clinical response or gastroesophageal reflux compared to SG, where up to 34% of patients require a conversion into a bypass procedure in up to 14 years of follow-up.^18^

Given the increasing number of interventions and advancements in surgical techniques, a comprehensive comparison of these modalities is warranted. While both surgical methods have well-documented benefits for obesity-related comorbidities, comparative long-term data on cardiovascular outcomes remain limited and often lack sufficient power in clinical trials.^19^

Therefore, this study aims to assess whether there is a difference in major adverse cardiac events (MACE) after GB vs SG. Building on a previous 7-year analysis,^20^ this study extends the follow-up period to 11 years and incorporates additional end points, including all-cause mortality.

## Methods

### Data Source and Study Design

This nationwide retrospective cohort study was conducted using administrative data from adults who underwent first-time GB or SG provided by the Federal Statistical Office in Switzerland (Bundesamt für Statistik) from January 2012 to December 2022.

The database includes all Swiss inpatient discharge records from acute care, general, and specialty hospitals in Switzerland. Individual-level data on patient demographics, health care utilization, hospital typology, medical diagnoses, diagnostic tests, clinical procedures, in-hospital patient outcomes, and date of death were provided for all hospitalized patients in Switzerland. An anonymized dataset was processed for the purpose of this analysis. Each hospitalization was unique, and all potential rehospitalizations were tracked and recorded. Medical diagnoses were coded by the *International Statistical Classification of Diseases and Related Health Problems, Tenth Revision, German Modification* (*ICD-10-GM* [hereafter *ICD-10*]) codes, and diagnostic and therapeutic interventions were coded by the Swiss procedural classification (CHOP) codes. The ethical review board of Northwestern Switzerland (Ethikkommission Nordwest und Zentralschweiz [EKNZ]) declared that this study did not fall under the scope of the Human Research Act, as data were anonymized before analysis (Req-2021-01397). This study followed the Strengthening the Reporting of Observational Studies in Epidemiology (STROBE) reporting guidelines.^21^

### Study Population and Exposure Definition

Patients aged 18 years or older classified as obese according to *ICD-10* codes for World Health Organization (WHO) obesity class I or higher (body mass index [BMI] >29.9, calculated as weight in kilograms divided by height in meters squared) and hospitalized for GB or SG were included in the study. Cohort entry was the first day of the index hospitalization (first-time metabolic surgery), excluding patients who had a CHOP code for a previous bariatric procedure (eg, bariatric conversion surgery, a surgical reintervention after a previous bariatric surgery, a biliopancreatic diversion, or any other surgery). Details on all *ICD-10* and CHOP codes used in this study are provided in eTable 1 in Supplement 1.

In accordance with the guidelines for surgery of morbid obesity set by the Swiss Society for the Study of Morbid Obesity and Metabolic Disorders and to reduce the risk of unmeasured confounding due to high cardiovascular recurrence risk, patients with a hospitalization for acute myocardial infarction (MI), ischemic stroke, or heart failure (HF) in the 6 months before cohort entry were excluded.^17^ To align with eligibility criteria of previous clinical trials,^22,23^ patients with end-stage kidney disease, dialysis-dependent kidney disease, or a primary diagnosis of cancer were also excluded. To address potential confounding related to different indications for gastric interventions, patients with any history of gastrointestinal cancers were also excluded (eTable 2 in Supplement 1). Additionally, patients admitted through the emergency department were not considered.

Except for in-hospital outcomes, the assessment of clinical outcomes started the day after hospital discharge and continued until the occurrence of a specific study outcome, death, or the end of study period on December 31, 2022, whichever came first.

### Outcomes

The primary composite outcome of 4-point MACE consisted of acute MI, ischemic stroke, HF, and all-cause mortality. Secondary outcomes of interest included the individual components of MACE, any revision surgery (any abdominal surgery potentially related to the index bariatric procedure but not directly affecting bariatric physiology), conversion surgery (any surgery modifying the index bariatric procedure), gastroesophageal reflux disease (GERD), hospitalization for dumping syndrome, and psychiatric disorders needing hospitalization.

Secondary short-term outcomes were all-cause in-hospital mortality and 30-day hospital readmissions. Immediate adverse events during the index hospitalization were also assessed, such as complications from surgical interventions; infections, including streptococcal or other sepsis; hypovolemic and septic shock; and digestive system complications following surgery, including postsurgical ileus, dumping syndrome, anastomotic leaks, strictures, and mechanical or infectious complications from gastrointestinal prosthetic devices. All *ICD-10* and CHOP codes on outcomes are available in eTable 3 in Supplement 1.

### Statistical Analysis

Descriptive statistics summarized baseline characteristics. Means and SDs were reported for continuous variables, while numbers, frequencies, and percentages were given for categorical variables. Standardized mean differences (SMDs) assessed group differences between the 2 surgical modalities; meaningful imbalances were defined as a SMD greater than 0.1.^24^

To address imbalances in patient characteristics between cohorts, the main analysis was conducted after creating a weighted pseudopopulation by inverse probability of treatment weighting (IPW). First, an exposure propensity score (PS) was calculated as the predicted probability of receiving GB or SG conditional upon all patient baseline covariates using a multivariable logistic regression model.^25^ To avoid extreme weights, we trimmed 2% of the most extreme PS. Additionally, crude and simple covariate-adjusted analyses were conducted. For detailed information on parameters included into the logistic model, see eTables 4 and 5 in Supplement 1. Age, which was only provided as a categorical parameter in 5-year intervals due to data privacy reasons, was further stratified into 3 groups. For all longitudinally assessed outcomes, unadjusted, simple covariate-adjusted, and weighted number of events, mean follow-up times, incidence rates (IR) per 1000 patient-years (PY), and hazard ratios (HR) with 95% confidence intervals using Cox regression models were calculated. For binary in-hospital outcomes, unadjusted, simple covariate-adjusted, and weighted number of events, IR per 1000 PY, and risk ratios (RR) with 95% confidence intervals were calculated using logistic regression.

To assess the robustness of our results, we also conducted a sensitivity analysis using 1:1 PS matching with the nearest-neighbor method with a maximum caliper of 0.001 on the propensity scale.^26^ Another sensitivity analysis excluded patients undergoing conversion surgeries, as we could not account for planned conversions, such as sleeve gastrectomy, as a preliminary step before definitive gastric bypass.

There were no missing data for patient characteristics and study outcomes. All tests were 2-sided, *P* < .05 was considered statistically significant, and 95% confidence intervals were reported for all effect measures. The statistical analyses were performed using Stata version 17.0 (StataCorp).

## Results

### Study Cohort and Patient Characteristics

Between July 2012 and December 2022, we identified 39 867 eligible patients, including 30 668 patients (76.9%) who underwent GB and 9199 patients (23.1%) who underwent SG (eTable 6 in Supplement 1). Compared with those undergoing GB, patients undergoing SG were older, more obese, more likely to be male, and had a higher burden of comorbidities, as shown by the Elixhauser comorbidity index.

After weighting, 39 067 patients were included in the main analysis, with 30 270 patients (77.5%) undergoing GB and 8797 patients (22.5%) undergoing SG (eFigure 1 in Supplement 1). Median (IQR) patient age was 42 (35-50) years, and 28 560 patients (73.1%) were women. A total of 23 708 patients (60.7%) had a body mass index (calculated as weight in kilograms divided by height in meters squared) of 40 or higher. Baseline characteristics of the weighted pseudopopulation were well balanced, and the PS distribution demonstrated good overlap before and after adjustment (eFigures 2 and 3 in Supplement 1). Detailed patient characteristics, demographic information, and comorbidities for both groups before and (for the pseudopopulations) after weighting are presented in Table 1.

**Table 1.** Baseline Characteristics Before and After Inverse Probability–Weighted (IPW) Balancing

Characteristic	Observed cohort before IPW			Balanced cohort after IPW^a^		
	No. (%)		SMD	No. (%)		SMD
	Gastric bypass (n = 30 668)	Sleeve gastrectomy (n = 9199)		Gastric bypass (n = 39 465.6)	Sleeve gastrectomy (n = 35 178.1)	
Demographics						
Age, y						
<40	13 462 (43.9)	3833 (41.7)	0.12	17 175.6 (43.5)	15 522.8 (44.1)	0.003
40-59	15 042 (49.0)	4411 (48.0)		19 189.6 (48.6)	16 768.8 (47.7)	
≥60	2164 (7.1)	955 (10.4)		3100.5 (7.9)	2886.5 (8.2)	
Sex						
Female	23 430 (76.4)	5896 (64.1)	0.27	28 903.1 (73.2)	24 911.0 (70.8)	0.05
Male	7238 (23.6)	3303 (35.9)		10 562.5 (26.8)	10 267.1 (29.2)	
Swiss citizen	22 825 (74.4)	6584 (71.6)	0.06	29 019.4 (73.5)	25 336.8 (72.0)	0.03
Supplementary insurance	3070 (10.0)	906 (9.8)	0.005	3923.5 (9.9)	3421.7 (9.7)	0.007
Admission to university hospital	2822 (9.2)	1378 (15.0)	−0.18	4198.3 (10.6)	40,63.2 (11.6)	−0.03
Burden of comorbidities						
Elixhauser comorbidity score, mean (SD)	1.7 (1.0)	2.0 (1.1)	−0.24	1.8 (1.0)	1.8 (1.0)	−0.04
Hospital frailty risk score						
<5	30 445 (99.3)	9089 (98.8)	0.05	39 110.6 (99.1)	34 865.7 (99.1)	−0.001
5-15	217 (0.7)	107 (1.2)		342.4 (0.9)	307.6 (0.9)	
>15	6 (0.0)	3 (0.0)		12.6 (0.0)	4.8 (0.0)	
Comorbidities						
Obesity classification WHO						
Obesity class I (BMI: 30.0-34.9)^b^	641 (2.1)	126 (1.4)	0.12	695.5 (1.8)	459.9 (1.3)	−0.009
Obesity class II (BMI: 35.0-39.9)^b^	11 351 (37.0)	2976 (32.4)		14 178.0 (35.9)	12 417.2 (35.3)	
Obesity class III (BMI ≥40.0)^b^	18 147 (59.2)	5898 (64.1)		23 885.6 (60.5)	21 684.5 (61.6)	
Unknown obesity class	529 (1.7)	199 (2.2)		706.5 (1.8)	616.5 (1.8)	
Type 1 diabetes	85 (0.3)	35 (0.4)	−0.02	120.5 (0.3)	126.8 (0.4)	−0.01
Type 2 diabetes	4042 (13.2)	1477 (16.1)	−0.08	5513.9 (14.0)	5046.0 (14.3)	−0.01
Chronic kidney disease	209 (0.7)	172 (1.9)	−0.11	378.4 (1.0)	372.0 (1.1)	−0.009
Hypertension	8762 (28.6)	3357 (36.5)	−0.17	12 096.5 (30.7)	11 299.4 (32.1)	−0.03
Congestive heart failure	86 (0.3)	57 (0.6)	−0.05	146.6 (0.4)	144.7 (0.4)	−0.006
Coronary heart disease	596 (1.9)	323 (3.5)	−0.10	925.4 (2.3)	906.0 (2.6)	−0.01
Atrial fibrillation	240 (0.8)	196 (2.1)	−0.11	437.8 (1.1)	435.2 (1.2)	−0.01
Cerebrovascular disease	21 (0.1)	10 (0.1)	−0.01	30.5 (0.1)	28.8 (0.1)	−0.002
Peripheral artery disease	38 (0.1)	27 (0.3)	−0.04	65.4 (0.2)	63.2 (0.2)	−0.003
COPD	383 (1.2)	214 (2.3)	−0.08	590.6 (1.5)	564.8 (1.6)	−0.008
OSAS	5182 (16.9)	2126 (23.1)	−0.16	7309.2 (18.5)	7028.7 (20.0)	−0.04
Dyslipidemia	3781 (12.3)	1392 (15.1)	−0.08	5153.2 (13.1)	4724.0 (13.4)	−0.01
Hepatopathy	1203 (3.9)	677 (7.4)	−0.15	1866.4 (4.7)	1820.7 (5.2)	−0.02
MASLD	1188 (3.9)	661 (7.2)	−0.15	1837.1 (4.7)	1791.0 (5.1)	−0.02
Osteoporosis	54 (0.2)	23 (0.3)	−0.02	76.8 (0.2)	61.9 (0.2)	0.004
Gastroesophageal reflux disease	6165 (20.1)	1370 (14.9)	0.14	7365.9 (18.7)	5977.9 (17.0)	0.04
Peptic ulcer disease	62 (0.2)	21 (0.2)	−0.006	81.4 (0.2)	78.7 (0.2)	−0.004
Solid cancer	34 (0.1)	15 (0.2)	0.01	47.6 (0.1)	46.1 (0.1)	−0.003
Psychiatric disorders						
Psychiatric disorders overall	3386 (11.0)	1228 (13.3)	0.07	4586.8 (11.6)	4155.5 (11.8)	−0.006
Depression	2283 (7.4)	809 (8.8)	0.05	3069.2 (7.8)	2828.0 (8.0)	−0.01
Anxiety and obsessive-compulsive disorders	390 (1.3)	138 (1.5)	0.02	525.2 (1.3)	494.5 (1.4)	−0.006
Substance abuse	292 (1.0)	109 (1.2)	0.02	401.4 (1.0)	330.6 (0.9)	0.008
Eating disorders	144 (0.5)	24 (0.3)	0.03	164.0 (0.4)	84.2 (0.2)	0.03
Type of surgery						
Proximal gastric bypass	18 138 (59.1)	NA	NA	24 207.6 (61.3)	NA	NA
Roux-en-Y gastric bypass	3824 (12.5)	NA	NA	5120.9 (13.0)	NA	NA
Distal gastric bypass	767 (2.5)	NA	NA	1020.2 (2.6)	NA	NA
Omega loop gastric bypass	308 (1.0)	NA	NA	412.4 (1.0)	NA	NA
Not otherwise specified gastric bypass	7638 (24.9)	NA	NA	8713.8 (22.1)	NA	NA

Abbreviations: BMI, body mass index; COPD, chronic obstructive pulmonary disease; MASLD, metabolic dysfunction–associated steatotic liver disease; NA, not applicable; OSAS, obstructive sleep apnea; SMD, standardized mean difference; WHO, World Health Organization.

^a^ Pseudopopulation estimates after trimming of 2% of most extreme weights (n = 800).

^b^ Calculated as weight in kilograms divided by height in meters squared.

### MACE

Over a median (IQR) follow-up period of of 5.1 years (2.6-7.6) years, 593 events (1.9%) were identified in the GB cohort and 276 events (3.0%) in the SG cohort, with event rates of 3.64 and 6.35 per 1000 PY, respectively. After adjustment, the risk of MACE was lower in the GB cohort compared to the SG cohort, with an adjusted HR of 0.83 (95% CI, 0.71-0.97) (eTable 7 in Supplement 1). Among the individual components of MACE, acute MI was observed less frequently in patients who underwent GB, with no differences otherwise between groups.

Following weighting, a total of 577 patients (1.9%) in the GB group and 264 (3.0%) in the SG group experienced MACE, corresponding to incidence rates of 3.96 and 5.10 per 1000 PY, respectively. The weighted HR for MACE in the GB cohort vs the SG cohort was 0.75 (95% CI, 0.64-0.88) (Table 2). These findings were driven by a lower event rate of acute MI (HR, 0.60; 95% CI, 0.43-0.82) in the GB cohort, while the risks of ischemic stroke, HF, and all-cause mortality were comparable between the groups.

**Table 2.** Primary and Secondary Long-Term Outcomes After Inverse Probability Weighting (IPW)^a^

Outcome	Gastric bypass			Sleeve gastrectomy			HR (95% CI)	*P* value
	No. (%)		IR/1000 PY	No. (%)		IR/1000 PY		
	Population (n = 30 270)	Pseudopopulation (n = 39 465.6)^b^		Population (n = 8797)	Pseudopopulation (n = 35 178.1)^b^			
Primary outcome								
MACE	577 (1.9)	802.5 (2.0)	3.96	264 (3.0)	858.4 (2.4)	5.10	0.75 (0.64-0.88)	<.001
Secondary outcomes								
Myocardial infarction	129 (0.4)	182.7 (0.5)	0.90	69 (0.8)	241.1 (0.7)	1.43	0.60 (0.43-0.82)	.002
Ischemic stroke	108 (0.4)	149.0 (0.4)	0.73	50 (0.6)	159.3 (0.5)	0.94	0.76 (0.54-1.08)	.13
Hospitalization for heart failure	69 (0.2)	105.2 (0.3)	0.52	40 (0.5)	107.1 (0.3)	0.63	0.79 (0.51-1.21)	.28
All-cause mortality	334 (1.1)	464.3 (1.2)	2.28	133 (1.5)	424.8 (1.2)	2.50	0.87 (0.70-1.09)	.23
Revision surgery^c^	2943 (9.7)	3754.8 (9.5)	19.68	224 (2.5)	914.8 (2.6)	5.47	3.63 (3.14-4.19)	<.001
Conversion surgery^d^	360 (1.2)	461.4 (1.2)	2.28	725 (8.2)	2933.2 (8.3)	18.32	0.13 (0.11-0.14)	<.001
GERD and peptic ulcer disease	2375 (7.8)	3087.9 (7.8)	16.00	916 (10.4)	3670.9 (10.4)	23.27	0.71 (0.65-0.77)	<.001
Hospitalization for dumping syndrome	270 (0.9)	336.7 (0.9)	1.66	43 (0.5)	180.6 (0.5)	1.07	1.56 (1.12-2.17)	.009
Psychiatric disorders and suicide attempt needing hospitalization	1872 (6.2)	2449.3 (6.2)	12.53	479 (5.4)	1855.9 (5.3)	11.37	1.12 (1.00-1.24)	.04

Abbreviations: GERD, gastroesophageal reflux disease; HR, hazard ratio; IR, incidence rate; MACE, major adverse cardiac event; PY, person-years.

^a^ Cox regression model comparing gastric bypass vs sleeve gastrectomy after IPW, adjusted for age, sex (female vs male), nationality (Swiss vs other nationality), residence before hospital admission (home, nursing home, psychiatric clinic, rehabilitation, penal institution, or not known), year of the index admission, length of hospital stay, hospital site (university hospital vs other hospitals), coronary heart disease, heart failure, atrial fibrillation, cerebrovascular disease, peripheral arterial vascular disease, cancer, chronic obstructive pulmonary disease, obstructive sleep apnea syndrome, chronic kidney disease, obesity, type 1 or 2 diabetes, arterial hypertension, dyslipidemia, hepatopathy, osteoporosis, psychiatric diseases, gastric banding, GERD, peptic ulcer disease, metabolic-associated fatty liver disease, and Elixhauser comorbidity index and frailty score.

^b^ Pseudopopulation estimates after IPW and 2% trimming of most extreme weights.

^c^ Revision meaning any abdominal operation potentially related to the index bariatric procedure but not directly affecting bariatric physiology, including pyloroplasty, revision gastroenteric anastomosis, Petersen space revision, implantation or change of a self-expanding endoprosthesis, surgery for hiatal hernia (abdominal or thoracic), and intraoperative manipulation of the stomach.

^d^ Conversion meaning any operation that involved modifying the index bariatric procedure, including biliopancreatic diversion, sleeve resection, resleeve resection (open or laparoscopic), additional gastrectomy after bariatric surgery (open or laparoscopic), gastric bypass, proximal gastric bypass after sleeve resection (reversal surgery, open or laparoscopic), distal gastric bypass (open or laparoscopic), omega-loop gastric bypass, and reinstallation of an intestinal anastomosis.

**Figure 1.**
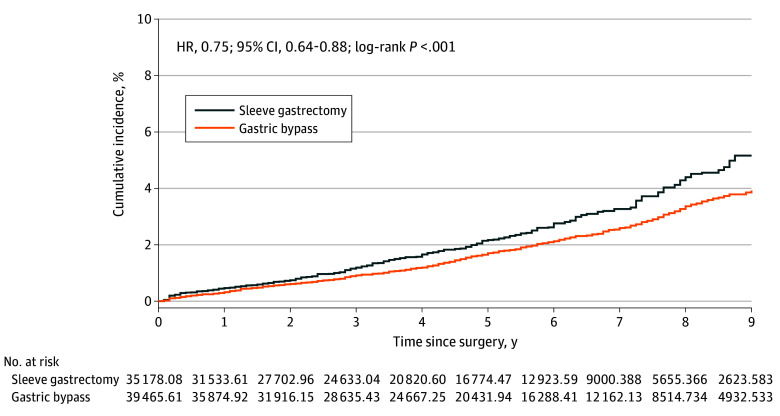
Cumulative Incidence of Major Adverse Cardiac Events (MACE) Inverse probability–weighted (IPW) Kaplan-Meier curves for MACE, with the follow-up truncated at 9 years postsurgery. Number at risk represents the number in the pseudopopulation generated by the IPW. HR indicates hazard ratio.

**Figure 2.**
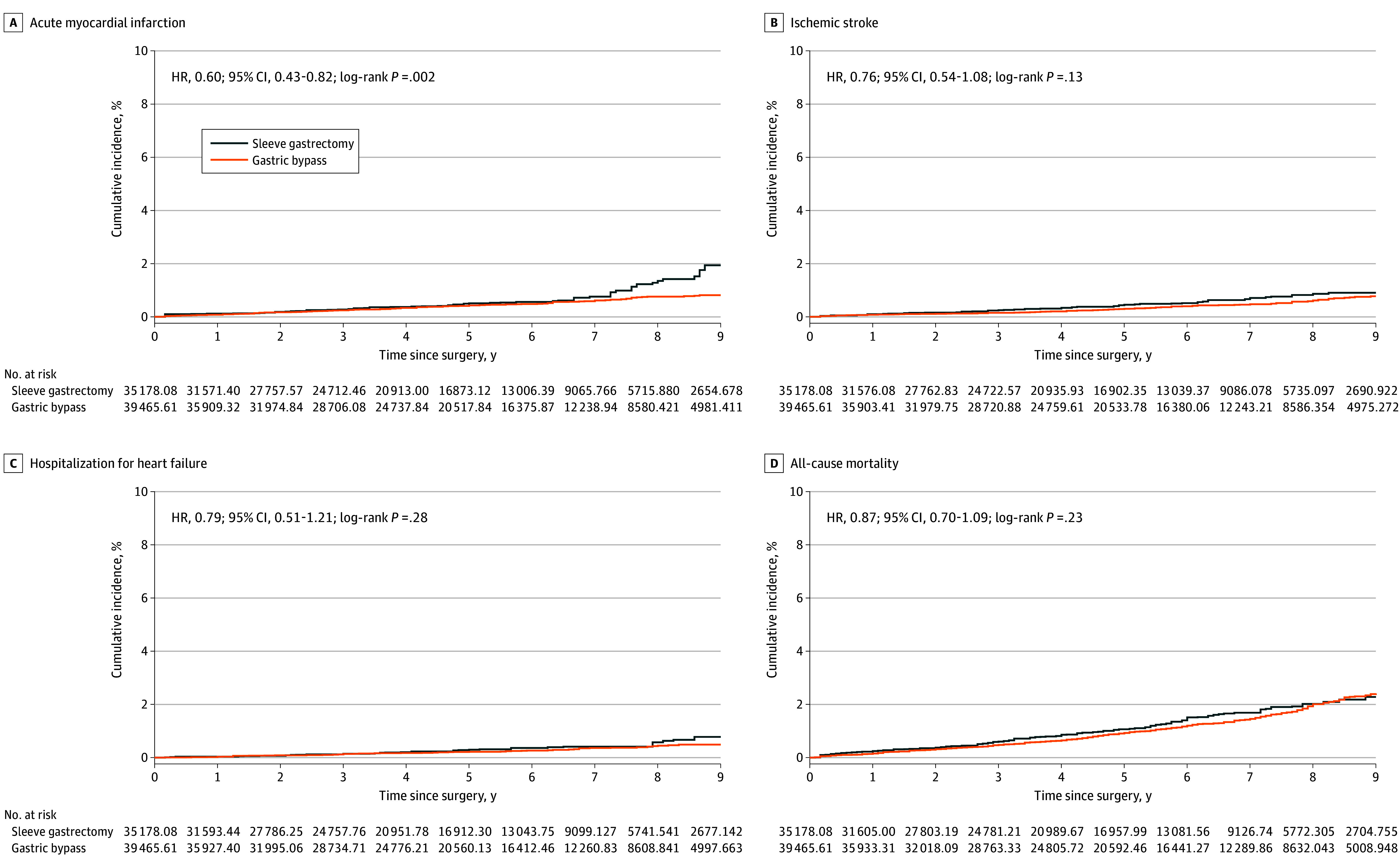
Cumulative Incidences of the Individual Components of Major Adverse Cardiac Events (MACE) Inverse probability–weighted (IPW) Kaplan-Meier curves of acute myocardial infarction (A), ischemic stroke (B), hospitalization for heart failure (C), and all-cause mortality (D), with the follow-up truncated at 9 years postsurgery. Number at risk represents the number in the pseudopopulation generated by the IPW. HR indicates hazard ratio.

The cumulative incidence depicted in the IPW Kaplan-Meier plots for MACE and its individual components was consistent with these findings (Figure 1 and Figure 2).

### Further Secondary Long-Term Outcomes

After weighting, consistent with the adjusted analysis, patients undergoing GB had a lower risk of conversion surgery (HR, 0.13; 95% CI, 0.11-0.14) and for GERD or peptic ulcer disease (HR, 0.71; 95% CI, 0.65-0.77) (Table 2). Conversely, patients in the GB group had an almost 4-fold increased risk of reoperation for revision of the initial surgery (HR, 3.63; 95% CI, 3.14-4.19) and were more likely to be hospitalized for dumping syndrome (HR, 1.56; 95% CI, 1.12-2.17) and for psychiatric disorders (HR, 1.12; 95% CI, 1.00-1.24) (Table 2). Crude and unweighted results for all outcomes are presented in eTables 7 and 8 in Supplement 1.

### In-Hospital Mortality, Immediate Complications, and Hospital Readmission

After adjustment, there were no differences in in-hospital mortality (adjusted RR, 0.56; 95% CI, 0.15-2.08), all-cause 30-day readmission (adjusted RR, 1.13; 95% CI, 1.00-1.27), or immediate in-hospital complications (adjusted RR, 1.07; 95% CI, 0.98-1.17) (eTable 8 in Supplement 1).

However, following IPW, while the risk of in-hospital mortality and all-cause 30-day readmission remained similar between interventions, GB was associated with a higher risk for immediate in-hospital complications (weighted RR, 1.25; 95% CI, 1.12-1.39) (Table 3).

**Table 3.** Secondary Short-Term Outcomes After Inverse Probability Weighting (IPW)^a^

Outcome	No. (%)				RR (95% CI)	*P* value
	Gastric bypass		Sleeve gastrectomy			
	Population (n = 30 270)	Pseudopopulation (n = 39 465.6)^b^	Population (n = 8797)	Pseudopopulation (n = 35 178.1)^b^		
In-hospital mortality	5 (0.02)	6.7 (0.02)	6 (0.07)	10.5 (0.03)	0.58 (0.17-1.93)	.39
All-cause 30-d readmission	1214 (4.0)	1618.9 (4.1)	353 (4.0)	1279.0 (3.6)	1.13 (1.00-1.28)	.05
Immediate postoperative complications during index hospitalization^c^	1654 (5.5)	2175.2 (5.5)	435 (4.9)	1555.9 (4.4)	1.25 (1.12-0.39)	<.001

Abbreviation: RR, risk ratio.

^a^ Logistic regression model comparing gastric bypass to sleeve gastrectomy after IPW, adjusted for age, sex (female vs male), nationality (Swiss vs other nationality), residence before hospital admission (home, nursing home, psychiatric clinic, rehabilitation, penal institution, or not known), year of the index admission, length of hospital stay, hospital site (university hospital vs other hospitals), coronary heart disease, heart failure, atrial fibrillation, cerebrovascular disease, peripheral arterial vascular disease, cancer, chronic obstructive pulmonary disease, obstructive sleep apnea syndrome, chronic kidney disease, obesity, type 1 or 2 diabetes, arterial hypertension, dyslipidemia, hepatopathy, osteoporosis, psychiatric diseases, gastric banding, gastroesophageal reflux disease, peptic ulcer disease, metabolic-associated fatty liver disease, and Elixhauser comorbidity index and frailty scores.

^b^ Pseudopopulation estimates after IPW and 2% trimming of most extreme weights.

^c^ Defined as complications of procedures, mechanical complications of gastrointestinal prosthetic devices, implants, grafts, infection, and inflammatory reaction due to internal gastrointestinal prosthetic devices, implants and grafts, complications of surgical and medical care, streptococcal sepsis, intraoperative and postprocedural complications and disorders of the digestive system, hypovolemic shock, and septic shock.

### Sensitivity Analyses

After 1:1 PS matching, a well-balanced distribution of baseline characteristics was achieved between the 2 groups, resulting in 9008 matched pairs each (eTable 9 in Supplement 1). While there was evidence suggesting a lower likelihood of 4-point MACE in patients undergoing GB, the difference between the groups did not reach statistical significance (HR, 0.92; 95% CI, 0.77-1.10) (eTable 10 in Supplement 1).

Another sensitivity analysis was conducted excluding 1144 patients who underwent conversion surgery, leaving 30 301 patients undergoing GB and 8422 patients undergoing SG (eTable 11 in Supplement 1). Baseline characteristics remained balanced after IPW, and the GB group consistently exhibited a lower rate of MACE compared to the SG group, consistent with the primary analysis (eTable 12 in Supplement 1).

## Discussion

This nationwide cohort study, which spans 11 years and encompasses over 39 000 patients in Switzerland, showed 2 key findings. First, GB was associated with a lower risk for MACE compared to SG, primarily due to lower rates of MI. Second, the safety outcomes—such as common complications, reoperations, and bariatric conversions—were consistent with existing literature. GB was linked to higher rates of short-term complications and revisions, while patients undergoing SG had a higher incidence of GERD and a greater likelihood of requiring conversion surgery due to suboptimal clinical response, GERD, or both.

The association of MBS with reduced macrovascular disease compared to medical treatment is well established.^27,28,29,30,31,32^ Most prior research has focused on end points like weight loss or type 2 diabetes remission,^12,33,34,35^ with less emphasis on how GB and SG impact cardiovascular events. The few observational studies, however, suggest that GB may offer superior cardiovascular outcomes compared to SG.^36,37^

In a recent study by our team,^20^ we observed a trend favoring GB for cardiovascular outcomes, although statistical significance was not reached. However, given the larger population and more extended follow-up of this study, our current analysis shows a lower risk of MACE in the GB group, adding to the growing evidence of its cardiovascular benefits over SG. Moreover, previous studies faced limitations, such as outdated data from the early SG era^37^ or broad definitions of MACE that included less specific end points.^38^

The largest randomized clinical trials comparing GB and SG, including the SM-BOSS-trial,^18,33^ the SLEEVEPASS trial,^39^ and the recent SleeveBypass trial,^40^ were either statistically underpowered to detect differences in MACE or did not focus on cardiovascular events as primary outcomes. However, with SG now being a globally standardized procedure supported by sufficient long-term data, our study addresses key limitations of earlier research, offering a clearer picture of the cardiovascular outcomes associated with these 2 widely performed surgeries.

The difference in MACE between GB and SG was primarily due to reduced rates of acute MI in the GB group. Interestingly, no differences in stroke incidence were observed. Along with slightly superior weight loss,^18,39,40,41^ GB led to more pronounced improvements in lipid profiles and cardiovascular risk factors.^8,33,42,43,44,45^ Furthermore, differences in hormonal changes between GB and SG are well documented, with GB inducing different and, to a certain extent, also greater changes in gut hormones and adipokines, such as more elevated levels of glucagon-like peptide-1 (GLP-1), peptide-YY-36, and cholecystokinin, but a smaller reduction in ghrelin compared to SG.^42,46,47^ These hormonal shifts may contribute to the beneficial effects of bariatric surgery on cardiac function,^48,49^ with studies on GLP-1 receptor agonists suggesting a protective role in reducing lipotoxicity and cardiac inflammation, further supporting the potential cardiovascular benefits of more elevated GLP-1 levels after GB.^50^ Thus, the more pronounced hormonal changes after GB could potentially translate into a greater reduction in cardiovascular events. However, without laboratory data, this remains speculative, and further studies are needed to confirm the impact of gut hormones on MACE in a comparative setting. The observed lower hazard of cardiac events in GB suggests a multifactorial mechanism that could include better glycemic control, greater hormonal changes, and improved cardiovascular risk factor reduction. Additionally, patients undergoing SG tended to be older, more obese, and had more comorbidities, factors that may have contributed to their higher risk of MACE, despite methodological target trial emulation strategies. Interestingly, weighted Kaplan-Meier curves for MACE only diverged after approximately 2.5 years, suggesting that cardiovascular benefits of GB may emerge later, thus explaining why earlier studies with shorter follow-up periods showed no significant difference.^34,36^

Hospitalization for heart failure was a rare outcome in both GB and SG groups, consistent with findings from Sundström and colleagues,^51^ likely due to the younger age and low prevalence of cardiac dysfunction in bariatric patients, along with rigorous preoperative assessments. Mortality rates were also low and similar between the groups, likely due to standardized perioperative care. A binational cohort study of more than 60 000 patients^52^ and a meta-analysis of 174 772 participants^53^ both found no significant difference in all-cause mortality between GB and SG, with MBS overall reducing mortality by 49.2% compared to usual care. Our findings align with previous studies reporting higher short-term complications and revision rates for GB compared to SG.^40^ The complex nature of GB, involving rerouting of the gastrointestinal tract and 2 anastomoses, contributes to increased risks of anastomotic leaks, internal hernias, dumping syndrome, and the need for revisional surgeries.^54^ Our data showed a higher risk of revision surgery and short-term postoperative complications following GB.

SG has gained popularity due to its perceived safety and shorter operating times,^55^ surpassing GB in recent years. However, recent reviews indicate a decline in SG usage since 2018,^56^ reflecting concerns over long-term outcomes like recurrent weight gain and new-onset GERD, leading to more frequent surgical conversions.^57^ Our findings support this trend, showing a higher risk for conversion surgery after SG. GERD remains a prominent complication associated with SG and is a well-known factor contributing to conversion surgeries.^33,58,59,60^ Consistent with our results, recent reviews have reported a substantial prevalence of de novo GERD following SG.^58,59^ Similar, Peterli and colleagues^33^ and Salminen and colleagues^39^ found a higher incidence of GERD and esophagitis in SG compared to GB. Although our data indicated lower GERD rates, this discrepancy may be due to underreporting related to the limitations of *ICD*-based diagnoses in hospital records and the fact that patients with known GERD or symptoms of acid reflux are less likely to be considered for SG, highlighting potential confounding by indication.

A major strength of this 11-year retrospective cohort study is the large patient sample and extended follow-up, which enhances generalizability and allows for robust comparisons to real-world outcomes. The use of advanced statistical methods ensures high internal validity by effectively adjusting for confounders, minimizing bias, and confirming result consistency. Additionally, including all-cause mortality and a reliable 4-point composite MACE end point offers a comprehensive evaluation of both short-term and long-term outcomes, facilitating meaningful comparisons with other studies.

### Limitations

This study has limitations. First, this study has an observational and retrospective nature, which precludes establishing causality and positions the findings primarily as hypothesis generating. Second, studying MACE in MBS populations is challenging due to these events’ relatively low incidence. As seen in the 1:1 PS-matched analysis, the reduction of the cohort to 18 016 patients resulted in the hazard ratio between the groups not reaching statistical significance anymore, most likely due to insufficient power to detect rare MACE outcomes. Although patients with obesity are at high risk for cardiovascular complications, such events typically manifest later in life after prolonged obesity, resulting in a lower event rate in the predominantly young bariatric population. Consequently, large cohort studies with observational designs are often necessary to achieve sufficient statistical power, and their findings should be validated through additional population-based studies and randomized clinical trials. Third, selection bias in the surgical allocation of patients at high risk toward SG is an important consideration. Observed baseline differences, such as older age, more morbidity, and higher obesity rates, may reflect clinical preferences toward less invasive procedures. Despite rigorous data cleaning, adjustments, and weighting, residual confounding due to coding inaccuracies and unmeasured data remains a concern in nationwide registries, although absence of early separation in Kaplan-Meier curves suggests minimization of residual confounding. Additionally, we were unable to identify a suitable instrumental variable in our dataset that met both relevance and exogeneity assumptions. Fourth, our results primarily reflect patients at low risk, without prior cardiovascular events. Furthermore, our analysis only applies to patient having a nonzero probability of undergoing either treatment. These factors should be considered when interpreting the generalizability of our results. Furthermore, the absence of information on socioeconomic status, ethnicity, and tobacco use limits the generalizability to other demographic subgroups and precludes assessment of these critical cardiovascular risk factors. Moreover, key clinical outcomes, such as remission of diabetes, hyperlipidemia, hypertension, weight loss, and quality of life, could not be assessed due to the unavailability of anthropometric measurements, laboratory values, and medication usage. Additionally, since our data are derived solely from hospitalizations, outcomes typically evaluated in outpatient settings remain unaccounted for.

## Conclusions

This cohort study demonstrates that GB is associated with a lower incidence of MACE compared to SG over an 11-year follow-up period. While both procedures are effective for weight loss and improving cardiovascular outcomes, GB may be associated with greater cardiovascular benefits. However, this must be balanced against its higher risk of postoperative complications and the need for surgical revisions, emphasizing the importance of individualized patient selection and shared decision-making in clinical practice.

## Notes

Supplement 1. eTable 1. CHOP-Codes and *ICD*-Codes on Inclusion Criteria eTable 2. CHOP-Codes and *ICD*-Codes on Exclusion Criteria eTable 3. CHOP-Codes and *ICD*-Codes on Outcomes eTable 4. CHOP-Codes and *ICD*-Codes of Baseline Characteristics eTable 5. Parameters Included Into Logistic Model to Calculate Propensity-Scores eTable 6. Annual Distribution of Gastric Bypass and Sleeve Gastrectomy Procedures, 2012 to 2022 eTable 7. Primary and Secondary Long-Term Outcomes Before IPW eTable 8. Short-Term Outcomes Before IPW eTable 9. Baseline Patient Characteristics After 1:1 Propensity-Score Matching eTable 10. Primary and Secondary Outcomes After 1:1 Propensity-Score Matching eTable 11. Baseline Patient Characteristics Without Conversion Surgery Cohort Before and After IPW eTable 12. Primary and Secondary Outcomes Without Conversion Surgery Cohort and After IPW eFigure 1. Flowchart eFigure 2. Density of Propensity-Scores Before Matching, According to Surgical Procedure eFigure 3. Density of Propensity-Scores After Matching, According to Surgical Procedure

Supplement 2. Data Sharing Statement
