# Supplementary material for: Major Adverse Cardiac Events After Gastric Bypass vs Sleeve Gastrectomy
Source: JAMA Surg. 2025 May 7;160(6):690–700. doi: 10.1001/jamasurg.2025.1065 (PMC12060020; doi:10.1001/jamasurg.2025.1065)
Supplement: Supplement 1. — eTable 1. CHOP-Codes and ICD-Codes on Inclusion Criteria eTable 2. CHOP-Codes and ICD-Codes on Exclusion Criteria eTable 3. CHOP-Codes and ICD-Codes on Outcomes eTable 4. CHOP-Codes and ICD-Codes of Baseline Characteristics eTable 5. Parameters Included Into Logistic Model to Calculate Propensity-Scores eTable 6. Annual Distribution of Gastric Bypass and Sleeve Gastrectomy Procedures, 2012 to 2022 eTable 7. Primary and Secondary Long-Term Outcomes Before IPW eTable 8. Short-Term Outcomes Before IPW eTable 9. Baseline Patient Characteristics After 1:1 Propensity-Score Matching eTable 10. Primary and Secondary Outcomes After 1:1 Propensity-Score Matching eTable 11. Baseline Patient Characteristics Without Conversion Surgery Cohort Before and After IPW eTable 12. Primary and Secondary Outcomes Without Conversion Surgery Cohort and After IPW eFigure 1. Flowchart eFigure 2. Density of Propensity-Scores Before Matching, According to Surgical Procedure eFigure 3. Density of Propensity-Scores After Matching, According to Surgical Procedure [file jamasurg-e251065-s001.pdf]

Supplemental Online Content

Wildisen S, Laager R, Struja T, et al. Major adverse cardiac events after gastric bypass vs sleeve gastrectomy. *JAMA Surgery*. Published online May 7, 2025. doi:10.1001/jamasurg.2025.1065

eTable 1. CHOP-Codes and *ICD*-Codes on Inclusion Criteria
eTable 2. CHOP-Codes and *ICD*-Codes on Exclusion Criteria
eTable 3. CHOP-Codes and *ICD*-Codes on Outcomes
eTable 4. CHOP-Codes and *ICD*-Codes of Baseline Characteristics
eTable 5. Parameters Included Into Logistic Model to Calculate Propensity-Scores
eTable 6. Annual Distribution of Gastric Bypass and Sleeve Gastrectomy Procedures, 2012 to 2022
eTable 7. Primary and Secondary Long-Term Outcomes Before IPW
eTable 8. Short-Term Outcomes Before IPW
eTable 9. Baseline Patient Characteristics After 1:1 Propensity-Score Matching
eTable 10. Primary and Secondary Outcomes After 1:1 Propensity-Score Matching
eTable 11. Baseline Patient Characteristics Without Conversion Surgery Cohort Before and After IPW
eTable 12. Primary and Secondary Outcomes Without Conversion Surgery Cohort and After IPW
eFigure 1. Flowchart
eFigure 2. Density of Propensity-Scores Before Matching, According to Surgical Procedure
eFigure 3. Density of Propensity-Scores After Matching, According to Surgical Procedure

This supplemental material has been provided by the authors to give readers additional information about their work.

© 2025 Wildisen et al. *JAMA Surgery*.

eTable 1. CHOP-Codes and *ICD*-Codes on Inclusion Criteria

CHOP	***Gastric bypass***
44.31.x	Gastric bypass<br>Proximal gastric bypass<br>Distal gastric bypass<br>Roux-en-Y gastric bypass<br>Omega-loop gastric bypass
44.39.11	Omega-loop gastric bypass
44.39.12	Omega-loop gastric bypass
42.54	Roux-en-Y gastric bypass, coded before 2017
44.38	Gastric bypass, laparoscopic, coded before 2015
44.39	Gastric bypass, open surgery, coded before 2015
CHOP	***Sleeve gastrectomy***
43.89.4	Sleeve resection
43.89.40	Sleeve resection, open surgery
43.89.41	Sleeve resection, laparoscopic
43.89.42	Sleeve resection after gastric banding removal, open surgery
43.89.43	Sleeve resection after gastric banding removal, laparoscopic
ICD-10	***Obesity***
E65	Localized obesity
E66	Obesity

Abbr.: ICD-10: International classification of diseases, Tenth Revision; CHOP: Swiss classification of surgical interventions; x: denotes wildcard characters

© 2025 Wildisen et al. *JAMA Surgery*.

eTable 2. CHOP-Codes and *ICD*-Codes on Exclusion Criteria

CHOP	***Description***
43.89.44	Re-sleeve resection, open surgery
43.89.45	Re-sleeve resection, laparoscopic
43.89.2	Biliopancreatic diversion
43.89.3	Biliopancreatic diversion with duodenal switch
43.89.5	Duodenal switch
43.99.1	Additional gastrectomy after bariatric surgery
44.31.13	Proximal gastric bypass after sleeve resection, reversal surgery, open
44.31.14	Proximal gastric bypass after sleeve resection, reversal surgery, laparoscopic
44.5x	Revision gastroenteric anastomosis
44.92	Intraoperative manipulation of the stomach (reposition of gastric volvulus)
44.99.5	Implantation or change of a self-expanding endoprosthesis
45.91.1	Reinstallation of a footpoint-anastomosis of the small intestine after bariatric surgery*, before 2017*
45.97	Reinstallation of a footpoint-anastomosis of the small intestine*, from 2017 on*
45.97.11	Reinstallation of a footpoint-anastomosis of the small intestine, open surgery
45.97.12	Reinstallation of a footpoint-anastomosis of the small intestine, laparoscopic
46.99.93	Repair of mesenteric gap, open surgery
46.99.94	Repair of mesenteric gap, laparoscopic
53.7	Surgery for diaphragmatic hernia, abdominal
53.8	Surgery for diaphragmatic hernia, thoracic
ICD-10	***Description***
Cx	Malignant neoplasm, *if listed as main diagnosis*
C15	Malignant neoplasm of esophagus
C16	Malignant neoplasm of stomach
C17	Malignant neoplasm of small intestine
C18	Malignant neoplasm of colon
C19	Malignant neoplasm of rectosigmoid junction
C20	Malignant neoplasm of rectum
C21	Malignant neoplasm of anus and anal canal
C22	Malignant neoplasm of liver and intrahepatic bile ducts
C23	Malignant neoplasm of gallbladder
C24	Malignant neoplasm of other and unspecified parts of biliary tract
C25	Malignant neoplasm of pancreas
C26	Malignant neoplasm of other and ill-defined digestive organs
N18.5	Chronic kidney disease, stage 5
T82.4	Other complication of vascular dialysis catheter
T85.71	Infection and inflammatory reaction due to peritoneal dialysis catheter
Z49	Encounter for care involving renal dialysis
Z99.2	Dependence on renal dialysis
Z98.0	Status after gastric bypass or intestinal anastomosis

Abbr.: ICD-10: International classification of diseases, Tenth Revision; CHOP: Swiss classification of surgical interventions; x: denotes wildcard characters

© 2025 Wildisen et al. *JAMA Surgery*.

eTable 3. CHOP-Codes and *ICD*-Codes on Outcomes

ICD-10	***Primary outcome MACE and individual MACE components***
I21	Acute myocardial infarction
I50	Heart failure
I11.0	Hypertensive heart disease with heart failure
I13.0	Hypertensive heart and chronic kidney disease with heart failure
I13.2	Hypertensive heart and chronic kidney disease with heart failure and with stage 5 chronic kidney disease, or end stage renal disease
I63	Cerebral infarction
I64	Stroke, not defined as hemorrhage or infarction
CHOP	***Revision surgery[a]: Postoperative complications needing reoperation***
44.2	Pyloroplasty
44.5x	Revision gastroenteric anastomosis
46.99.93	Mesenteric gap revision with internal herniation, open surgery
46.99.94	Mesenteric gap revision with internal herniation, laparoscopic
44.99.5	Implantation or change of a self-expanding endoprosthesis
53.7	Surgery for diaphragmatic hernia, abdominal
53.8	Surgery for diaphragmatic hernia, thoracic
44.92	Intraoperative manipulation of the stomach (reposition of gastric volvulus)
CHOP	***Conversion surgery after index-hospitalization[b]***
43.89.2	Biliopancreatic diversion
43.89.3	Biliopancreatic diversion with duodenal switch (BPD/DS)
43.89.4	Sleeve resection
43.89.44	Re-sleeve resection, open surgery
43.89.45	Re-sleeve resection, laparoscopic
43.89.5	Duodenal Switch
43.99.10	Additional gastrectomy after bariatric surgery, open surgery
43.99.11	Additional gastrectomy after bariatric surgery, laparoscopic
44.31	Gastric bypass
44.31.13	Proximal gastric bypass after sleeve resection, reversal surgery, open surgery
44.31.14	Proximal gastric bypass after sleeve resection, reversal surgery, laparoscopic
44.31.21	Distal gastric bypass, open surgery
44.31.22	Distal gastric bypass, laparoscopic
44.31.3	Omega-loop gastric bypass
45.91.1	Reinstallation of a distal intestinal anastomosis of the small intestine after bariatric surgery, before 2017
45.97.	Reinstallation of a distal intestinal anastomosis of the small intestine
ICD-10	***GERD and peptic ulcer disease***
K21	Gastro-oesophageal reflux disease
K22.1	Ulcer of oesophagus
K25	Gastric ulcer
K26	Duodenal ulcer
K27	Peptic ulcer, site unspecified
K28	Gastrojejunal ulcer
ICD-10	***Hospitalization for dumping syndrome***
K91.1	Postgastric surgery syndromes (dumping syndrome)

© 2025 Wildisen et al. *JAMA Surgery*.

ICD-10	***Psychiatric disorder needing hospitalization and suicide attempt, only if in main diagnosis***
Fx	Psychiatric disorder
X84	Intentional self-harm
R45.8	Other symptoms and signs involving emotional state (Suicidal ideation)
ICD-10	***Immediate complications during index-hospitalization***
T81	Complications of procedures, not elsewhere classified
T85.5[c]	Mechanical complication of gastrointestinal prosthetic devices, implants and grafts
T85.6[c]	Mechanical complication of other specified internal and external prosthetic devices, implants and grafts
T85.76[c]	Infection and inflammatory reaction due to other internal gastrointestinal prosthetic devices, implants and grafts
T88.8	Other specified complications of surgical and medical care, not elsewhere classified
A40	Streptococcal sepsis
A41	Other sepsis
K91	Intraoperative and postprocedural complications and disorders of digestive system, not elsewhere classified
R57.1	Hypovolemic shock
R57.2	Septic shock

[a]Revisions are defined by any abdominal operation potentially related to the index bariatric procedure, but not directly affecting its bariatric physiology. C, Revisions included any operation that involved modifying the index bariatric procedure.
[b]Conversions included any operation that involved modifying the index bariatric procedure and its physiology.
[c]T85.x is only considered if listed as secondary diagnosis since as main diagnosis it is mostly used for revision of gastric banding and conversion in gastric bypass/sleeve gastrectomy
Abbr.: ICD-10: International classification of diseases, Tenth Revision; CHOP: Swiss classification of surgical interventions; x: denotes wildcard characters; MACE, major adverse cardiac event; GERD, Gastroesophageal reflux disease

© 2025 Wildisen et al. *JAMA Surgery*.

eTable 4. CHOP-Codes and *ICD*-Codes of Baseline Characteristics

ICD-10	***Obesity***
E65	Localized obesity
E66	Obesity
E66.00 /.10 /.20 /.80 /.90	Obesity WHO grade I
E66.01/.11 /.21 /.81 /.91	Obesity WHO grade II
E66.02/.12 /.22 /.82 /.92	Obesity WHO grade III
ICD-10	***Type 1 and Type 2 diabetes***
E10	Type 1 diabetes
E11	Type 2 diabetes
ICD-10	***Chronic kidney disease***
N18	Chronic kidney disease
N18.1	Chronic kidney disease, stage 1
N18.2	Chronic kidney disease, stage 2 (mild)
N18.3	Chronic kidney disease, stage 3 (moderate)
N18.4	Chronic kidney disease, stage 4 (severe)
N19	Unspecified kidney failure
ICD-10	***Hypertension***
I10	Essential (primary) hypertension
I11	Hypertensive heart disease
I12	Hypertensive chronic kidney disease
I13	Hypertensive heart and chronic kidney disease
I15	Secondary hypertension
I67.4	Hypertensive encephalopathy
ICD-10	***Congestive heart failure***
I11.0	Hypertensive Heart disease with heart failure
I13.0	Hypertensive heart and chronic kidney disease with heart failure
I13.2	Hypertensive heart and chronic kidney disease with heart failure and with stage 5 chronic kidney disease, or end stage renal disease
ICD-10	***Cardiovascular disease***
I20	Angina pectoris
I21	Acute myocardial infarction
I22	Subsequent ST elevation (STEMI) and non-ST elevation (NSTEMI) myocardial infarction
I23	Certain current complications following ST elevation (STEMI) and non-ST elevation (NSTEMI) myocardial infarction (within the 28 day period)
I24	Other acute ischemic heart diseases
I25	Chronic ischemic heart disease
ICD-10	***Atrial fibrillation***
I48	Atrial fibrillation
ICD-10	***Cerebrovascular disease***
I60	Nontraumatic subarachnoid hemorrhage
I61	Other and unspecified nontraumatic intracranial hemorrhage
I62	Certain current complications following ST elevation (STEMI) and non-ST elevation (NSTEMI) myocardial infarction (within the 28 day period)
I63	Cerebral infarction
I64	Family history of stroke

© 2025 Wildisen et al. *JAMA Surgery*.

I65	Occlusion and stenosis of precerebral arteries, not resulting in cerebral infarction
I66	Occlusion and stenosis of cerebral arteries, not resulting in cerebral infarction
I67	Other cerebrovascular diseases
I69	Sequelae of cerebrovascular disease
ICD-10	***Peripheral vascular disorder***
I70.2	Atherosclerosis of native arteries of the extremities
ICD-10	***Chronic obstructive pulmonary disease***
J44	Chronic obstructive pulmonary disease
ICD-10	***Obstructive sleep apnea***
E47.3	Sleep apnea
ICD-10	***Dyslipidemia***
E78	Disorders of lipoprotein metabolism and other lipidemias
ICD-10	***Hepatopathy and metabolic dysfunction-associated steatotic liver disease***
K70 - 77	Diseases of liver
K74	Fibrosis and cirrhosis of liver
K74.0	Hepatic fibrosis
K74.1	Hepatic sclerosis
K74.2	Hepatic fibrosis with hepatic sclerosis
K74.6	Other and unspecified cirrhosis of liver
K75	Other inflammatory liver disease
K75.8	Other specified inflammatory liver disease
K75.9	Inflammatory liver disease, unspecified
K76	Other disease of liver
ICD-10	***Osteoporosis***
M80	Osteoporosis with current pathological fracture
M81	Osteoporosis without current pathological fracture
M82	Osteoporosis, unspecified
ICD-10	***Gastroesophageal reflux disease and peptic ulcer disease***
K21	Gastro-esophageal reflux disease
K25	Gastric ulcer
K26	Gastro-duodenal ulcer
K27	Peptic ulcer, unspecified
K28	Gastrojejunal ulcer
CHOP	***Gastroesophageal reflux disease and peptic ulcer disease***
44.40	Surgical repair of peptic ulcer, unspecified
44.41	Surgical repair of gastric ulcer
44.42	Surgical repair of duodenal ulcer
ICD-10	***Solid cancer***
C0 – C7	Malignant neoplasm
ICD-10	***Psychiatric disorders***
Fx	Psychiatric disorders overall
F30	Manic episode
F32	Major depressive disorder, single episode
F33	Major depressive disorder, recurrent
F41	Other anxiety disorder

© 2025 Wildisen et al. *JAMA Surgery*.

F42	Obsessive-compulsive disorder
F55	Abuse of non-psychoactive substances
F1x	Substance-abuse related disorders
CHOP	***Type of surgery***
44.31.1	Proximal gastric bypass
44.31.4	Roux-en-Y gastric bypass
42.54	Roux-en-Y gastric bypass, coded before 2017
44.31.2	Distal gastric bypass
44.31.3	Omega-loop gastric bypass
44.39.11	Omega-loop gastric bypass, open surgery
44.39.12	Omega-loop gastric bypass, laparoscopic

Abbr.: ICD-10: International classification of diseases, Tenth Revision; CHOP: Swiss classification of surgical interventions; x: denotes wildcard characters; WHO: World Health Organization

© 2025 Wildisen et al. *JAMA Surgery*.

eTable 5. Parameters Included Into Logistic Model to Calculate Propensity-Scores

Outcome	Binary variables			Categorial variables	Continuous variables
MACE	Female gender	Peripheral artery disease	Hematologic malignancy	Obesity classification	Frailty score
	Swiss-nationality	Cerebrovascular disease	Depression	Chronic kidney disease classified	Length of stay
	Supplementary insurance	Congestive heart failure	Anxiety and obsessive-compulsive disorder	Year of treatment	Elixhauser comorbidity score
	Admission to university hospital	Atrial fibrilliation	Substance abuse	Age[a]	
	Admission from home	Hypertension	Eating disorder		
	Chronic obstructive pulmonary disease	Dyslipidemia	Psychiatric disorders overall		
	Coronary artery disease	Obstructive sleep apnea	Prior gastric banding		
	Type 1 diabetes	Hepatopathy	Gastroesophageal reflux disease		
	Type 2 diabetes	Metabolic dysfunction-associated steatotic liver disease	Peptic ulcer disease		
	Osteoporosis	Solid malignancy			

All 36 covariates included in the PS model. For the IPW analysis 2% of the data was trimmed of the extremes. For the 1 : 1 PS-matching the nearest neighbor method was used with a caliper of 0.001 on the PS scale. A standardized mean difference less than 0.10 was considered adequate for covariate balance after IPW and PS-matching.
[a]Age categorized in 5-year intervals (total of 17 groups) and stratified in 3 groups (<40years, 40-59years, ≥60years)
Abbr.: MACE: Major adverse cardiac event

© 2025 Wildisen et al. *JAMA Surgery*.

eTable 6. Annual Distribution of Gastric Bypass and Sleeve Gastrectomy Procedures, 2012 to 2022

	Gastric bypass	Sleeve gastrectomy
	**N=30,668**	**N=9,199**
**Year of intervention, n (%)**		
2012	1,342 (4.4)	206 (2.2)
2013	3,011 (9.8)	508 (5.5)
2014	3,136 (10.2)	677 (7.4)
2015	2,715 (8.9)	891 (9.7)
2016	3,100 (10.1)	1,057 (11.5)
2017	3,092 (10.1)	1,038 (11.3)
2018	3,236 (10.6)	1,005 (10.9)
2019	3,024 (9.9)	966 (10.5)
2020	2,434 (7.9)	792 (8.6)
2021	2,859 (9.3)	1,005 (10.9)
2022	2,719 (8.9)	1,054 (11.5)

© 2025 Wildisen et al. *JAMA Surgery*.

eTable 7. Primary and Secondary Long-Term Outcomes Before IPW

	Before IPW							
	**Gastric bypass**		**Sleeve gastrectomy**		**Crude**		**Adjusted**	
	**N=30,668**		**N=9,199**					
**Outcome**	N (%)	IR/1,000 PY	N (%)	IR/1,000 PY	HR (95% CI)	P	HR (95% CI)	P
Primary outcome								
MACE	593 (1.9)	3.64	276 (3.0)	6.35	0.55 (0.47 to 0.63)	<0.001	0.83 (0.71 to 0.97)	0.02
Secondary outcomes								
Myocardial infarction	132 (0.4)	0.81	73 (0.8)	1.67	0.46 (0.35 to 0.62)	<0.001	0.66 (0.49 to 0.90)	0.009
Ischemic stroke	110 (0.4)	0.67	53 (0.6)	1.21	0.53 (0.38 to 0.74)	<0.001	0.80 (0.57 to 1.14)	0.22
Hospitalization for heart failure	69 (0.2)	0.42	40 (0.4)	0.91	0.44 (0.30 to 0.65)	<0.001	1.01 (0.66 to 1.55)	0.97
All-cause mortality	345 (1.1)	2.11	140 (1.5)	3.19	0.62 (0.51 to 0.76)	<0.001	0.94 (0.76 to 1.16)	0.57
Revision surgery[a]	3016 (9.8)	19.71	248 (2.7)	5.75	3.49 (3.07 to 3.97)	<0.001	3.38 (2.97 to 3.86)	<0.001
Conversion surgery[b]	367 (1.2)	2.26	777 (8.4)	18.81	0.12 (0.11 to 0.14)	<0.001	0.12 (0.11 to 0.14)	<0.001
GERD and peptic ulcer disease	2422 (7.9)	15.63	986 (10.7)	24.28	0.67 (0.63 to 0.73)	<0.001	0.70 (0.64 to 0.75)	<0.001
Hospitalization for dumping syndrome	281 (0.9)	1.72	45 (0.5)	1.03	1.67 (1.22 to 2.29)	0.001	1.51 (1.09 to 2.08)	0.01
Psychiatric disorders and suicide attempt needing hospitalization	1910 (6.2)	12.16	504 (5.5)	11.96	1.03 (0.94 to 1.14)	0.50	1.17 (1.06 to 1.29)	0.003

Abbr.: IPW: Inverse probability weighting; N: number; IR: incidence rate; PY: person-years; CI: confidence interval; HR: hazard ratio; P: P-value; MACE: major adverse cardiac event; GERD: gastroesophageal reflux disease

[a]Revision meaning any abdominal operation potentially related to the index bariatric procedure but not directly affecting bariatric physiology, including Pyloroplasty, revision gastroenteric anastomosis,
Petersen's space revision, implantation or change of a self-expanding endoprosthesis, surgery for hiatal hernia (abdominal/thoracic), and intraoperative manipulation of the stomach.

[b]Conversion meaning any operation that involved modifying the index bariatric procedure, including Biliopancreatic diversion, sleeve resection, resleeve resection (open/laparoscopic), additional gastrectomy after bariatric surgery (open/laparoscopic), gastric bypass, proximal gastric bypass after sleeve resection (reversal surgery, open/laparoscopic), distal gastric bypass (open/laparoscopic), omega-loop gastric bypass, and reinstallation of an intestinal anastomosis.

Cox regression model comparing gastric bypass vs. sleeve gastrectomy, crude and adjusted for age, sex (female vs. male), nationality (Swiss vs. other nationality), residence before hospital admission (home, nursing home, psychiatric clinic, rehabilitation, penial institution, not known), year of the index-admission, length of hospital stay, hospital site (university hospital vs. other hospitals), coronary heart disease (CHD), heart failure (HF), atrial fibrillation (AFib), cerebrovascular disease, peripheral arterial vascular disease (PAVK), cancer, chronic obstructive pulmonary disease (COPD), obstructive sleep apnea syndrome (OSAS), chronic kidney disease (CKD), obesity, diabetes mellitus type 1 or 2, arterial hypertension, dyslipidemia, hepatopathy, osteoporosis, psychiatric diseases, gastric banding, gastroesophageal reflux disease, peptic ulcer disease, metabolic associated fatty liver disease, Elixhauser comorbidity index and frailty scores.

© 2025 Wildisen et al. *JAMA Surgery*.

eTable 8. Short-Term Outcomes Before IPW

	Before IPW					
	**Gastric bypass**	**Sleeve gastrectomy**	**Crude**		**Adjusted**	
	**N=30,668**	**N=9,199**				
**Outcome**	N (%)	N (%)	RR (95% CI)	P	RR (95% CI)	P
In-hospital mortality	5 (0.02)	6 (0.07)	0.25 (0.08 to 0.82)	0.08	0.56 (0.15 to 2.08)	0.43
All-cause 30-day readmission	1230 (4.0)	363 (3.9)	1.02 (0.91 to 1.14)	0.78	1.13 (1.00 to 1.27)	0.04
Immediate postoperative complications during index-hospitalization[a]	1770 (5.8)	486 (5.3)	1.09 (0.99 to 1.20)	0.07	1.07 (0.98 to 1.17)	0.14

Abbr.: IPW: Inverse probability weighting N: Number; RR: risk ratio; CI: Confidence interval; P: P-value

[a]Defined as complications of procedures, mechanical complications of gastrointestinal prosthetic devices, implants, grafts, infection, and inflammatory reaction due to internal gastrointestinal prosthetic devices, implants and grafts, complications of surgical and medical care, streptococcal sepsis, intraoperative and postprocedural complications and disorders of the digestive system, hypovolemic shock, and septic shock.

Logistic regression model comparing gastric bypass to sleeve gastrectomy, crude and adjusted for age, sex (female vs. male), nationality (Swiss vs. other nationality), residence before hospital admission (home, nursing home, psychiatric clinic, rehabilitation, penial institution, not known), year of the index-admission, length of hospital stay,, hospital site (university hospital vs. other hospitals), coronary heart disease (CHD), heart failure (HF), atrial fibrillation (AFib), cerebrovascular disease, peripheral arterial vascular disease (PAVK), cancer, chronic obstructive pulmonary disease (COPD), obstructive sleep apnea syndrome (OSAS), chronic kidney disease (CKD), obesity, type 1 or 2 diabetes, arterial hypertension, dyslipidemia, hepatopathy, osteoporosis, psychiatric diseases, gastric banding, gastroesophageal reflux disease, peptic ulcer disease, metabolic associated fatty liver disease,, Elixhauser comorbidity index and frailty scores

© 2025 Wildisen et al. *JAMA Surgery*.

eTable 9. Baseline Patient Characteristics After 1:1 Propensity-Score Matching

	Gastric bypass	Sleeve gastrectomy	SMD
	N=9,008	N=9,008	
**Demographics**			
Age, n (%)			0.09
< 40 years	3,393 (37.7)	3,812 (42.3)	
40-59 years	4,687 (52.0)	4,343 (48.2)	
> 60 years	928 (10.3)	853 (9.5)	
Female sex, n (%)	5,745 (63.8)	5,856 (65.0)	-0.03
Swiss citizen, n (%)	6,314 (70.1)	6,447 (71.6)	-0.03
Supplementary insurance, n (%)	1,075 (11.9)	885 (9.8)	0.07
Admission to university hospital, n (%)	1,312 (14.6)	1,276 (14.2)	0.01
**Burden of comorbidities**			
Elixhauser comorbidity score, mean (SD)	2.0 (1.1)	1.9 (1.1)	0.09
Hospital frailty risk score, n (%)			0.02
<5	8,896 (98.8)	8,918 (99.0)	
5-15	108 (1.2)	88 (1.0)	
>15	4 (0.0)	2 (0.0)	
**Comorbidities, n (%)**			
Obesity classification WHO			0.05
Obesity class I (BMI 30.0 - 34.9 kg/m2)	165 (1.8)	124 (1.4)	
Obesity class II (BMI 35.0 - 39.9 kg/m2)	3,031 (33.6)	2,942 (32.7)	
Obesity class III (BMI ≥ 40.0 kg/m2)	5,610 (62.3)	5,753 (63.9)	
Unknown obesity class	202 (2.2)	189 (2.1)	
Type 1 diabetes	36 (0.4)	33 (0.4)	0.005
Type 2 diabetes	1,614 (17.9)	1,395 (15.5)	0.07
Chronic kidney disease	119 (1.3)	117 (1.3)	0.002
Hypertension	3,577 (39.7)	3,205 (35.6)	0.09
Congestive heart failure	43 (0.5)	44 (0.5)	0.002
Coronary heart disease	316 (3.5)	279 (3.1)	0.02
Atrial fibrillation	149 (1.7)	149 (1.7)	<0.001
Cerebrovascular disease	8 (0.1)	9 (0.1)	0.004
Peripheral artery disease	21 (0.2)	19 (0.2)	0.004
COPD	187 (2.1)	184 (2.0)	0.002

© 2025 Wildisen et al. *JAMA Surgery*.

OSAS	2,128 (23.6)	2,017 (22.4)	0.03
Dyslipidemia	1,501 (16.7)	1,313 (14.6)	0.06
Hepatopathy	643 (7.1)	615 (6.8)	0.01
MASLD	629 (7.0)	603 (6.7)	0.01
Osteoporosis	22 (0.2)	21 (0.2)	0.002
Gastroesophageal reflux disease	1,538 (17.1)	1,365 (15.2)	0.05
Peptic ulcer disease	26 (0.3)	18 (0.2)	0.02
Solid cancer	16 (0.2)	13 (0.1)	0.008
**Psychiatric disorders, n (%)**			
Psychiatric disorders overall	1,304 (14.5)	1,172 (13.0)	0.04
Depression	870 (9.7)	776 (8.6)	0.04
Anxiety and obsessive-compulsive disorders	145 (1.6)	134 (1.5)	0.009
Substance abuse	122 (1.4)	105 (1.2)	0.02
Eating disorders	28 (0.3)	24 (0.3)	0.008
**Type of surgery, n (%)**			
Proximal gastric bypass	5,914 (65.7)	n.a.	n.a.
Roux-en-Y gastric bypass	1,258 (14.0)	n.a.	n.a.
Distal gastric bypass	264 (2.9)	n.a.	n.a.
Omega-loop gastric bypass	102 (1.1)	n.a.	n.a.
Not otherwise specified gastric bypass	1,472 (16.3)	n.a.	n.a.

Data are presented as mean (±SD) for continuous measures, and n (%) for categorical measures.
Abbr.: SD: Standard deviation; SMD: Standardized mean difference; n: Number; WHO: World Health Organization; BMI: Body-mass index (calculated as weight in kilograms divided by height in meters squared); COPD: Chronic obstructive pulmonary disease; OSAS: Obstructive sleep apnea; MASLD: Metabolic dysfunction-associated steatotic liver disease; n.a.: Not applicable.

© 2025 Wildisen et al. *JAMA Surgery*.

eTable 10. Primary and Secondary Outcomes After 1:1 Propensity-Score Matching

	After propensity-score matching					
	Gastric bypass		Sleeve gastrectomy			
	N=9,008		N=9,008			
**Outcome**	N (%)	IR/1,000 PY	N (%)	IR/1,000 PY	HR (95% CI)	P
Primary outcome						
MACE	229 (2.5)	5.29	245 (2.7)	5.73	0.92 (0.77 to 1.10)	0.36
Secondary outcomes						
Myocardial infarction	51 (0.6)	1.17	69 (0.8)	1.61	0.73 (0.51 to 1.04)	0.08
Ischemic stroke	46 (0.5)	1.06	51 (0.6)	1.19	0.89 (0.60 to 1.33)	0.57
Hospitalization for heart failure	30 (0.3)	0.69	31 (0.3)	0.72	0.95 (0.58 to 1.57)	0.85
All-cause mortality	127 (1.4)	2.92	120 (1.3)	2.78	1.04 (0.81 to 1.34)	0.75
Revision surgery[a]	804 (8.9)	19.68	243 (2.7)	5.73	3.43 (2.97 to 3.95)	<0.001
Conversion surgery[b]	115 (1.3)	2.66	765 (8.5)	18.84	0.14 (0.12 to 0.17)	<0.001
GERD and peptic ulcer disease	742 (8.2)	18.08	966 (10.7)	24.20	0.75 (0.68 to 0.83)	<0.001
Hospitalization for dumping syndrome	66 (0.7)	1.52	44 (0.5)	1.02	1.49 (1.01 to 2.18)	0.04
Psychiatric disorders and suicide attempt needing hospitalization	574 (6.4)	13.76	492 (5.5)	11.88	1.16 (1.03 to 1.31)	0.02

Abbr.: N: number; IR: incidence rate; PY: person-years; CI: confidence interval; HR: hazard ratio; P: P-value; MACE: major adverse cardiac event; GERD: gastroesophageal reflux disease

[a]Revision meaning any abdominal operation potentially related to the index bariatric procedure but not directly affecting bariatric physiology, including Pyloroplasty, revision gastroenteric anastomosis, Petersen's space revision, implantation or change of a self-expanding endoprosthesis, surgery for hiatal hernia (abdominal/thoracic), and intraoperative manipulation of the stomach.

[b]Conversion meaning any operation that involved modifying the index bariatric procedure, including Biliopancreatic diversion, sleeve resection, resleeve resection (open/laparoscopic), additional gastrectomy after bariatric surgery (open/laparoscopic), gastric bypass, proximal gastric bypass after sleeve resection (reversal surgery, open/laparoscopic), distal gastric bypass (open/laparoscopic), omega-loop gastric bypass, and reinstallation of an intestinal anastomosis.

Cox regression model comparing gastric bypass vs. sleeve gastrectomy, adjusted for age, sex (female vs. male), nationality (Swiss vs. other nationality), residence before hospital admission (home, nursing home, psychiatric clinic, rehabilitation, penial institution, not known), year of the index-admission, length of hospital stay, hospital site (university hospital vs. other hospitals), coronary heart disease (CHD), heart failure (HF), atrial fibrillation (AFib), cerebrovascular disease, peripheral arterial vascular disease (PAVK), cancer, chronic obstructive pulmonary disease (COPD), obstructive sleep apnea syndrome (OSAS), chronic kidney disease (CKD), obesity, type 1 or 2 diabetes, arterial hypertension, dyslipidemia, hepatopathy, osteoporosis, psychiatric diseases, gastric banding, gastroesophageal reflux disease, peptic ulcer disease, metabolic associated fatty liver disease, Elixhauser comorbidity index and frailty scores
.

© 2025 Wildisen et al. *JAMA Surgery*.

eTable 11. Baseline Patient Characteristics Without Conversion Surgery Cohort Before and After IPW

	Observed cohort before IPW			Balanced cohort after IPW[a]		
	Gastric bypass	Sleeve gastrectomy	SMD	Gastric bypass	Sleeve gastrectomy	SMD
	N=30,301	N=8,422		N=38,332.2	N=33,760.1	
**Demographics**						
Age, n (%)			0.12			0.003
< 40 years	13,280 (43.8)	3,532 (41.9)		16,700.9 (43.6)	14,923.6 (44.2)	
40-59 years	14,881 (49.1)	4,007 (47.6)		18,632.1 (48.6)	16,077.8 (47.6)	
≥ 60 years	2,140 (7.1)	883 (10.5)		2,999.1 (7.8)	2,758.7 (8.2)	
Female sex, n (%)	23,155 (76.4)	5,365 (63.7)	0.28	28,109.6 (73.3)	23,848.2 (70.6)	0.05
Swiss citizen, n (%)	22,545 (74.4)	6,021 (71.5)	0.07	28,185.1 (73.5)	24,258.3 (71.9)	0.04
Supplementary insurance, n (%)	3,025 (10.0)	851 (10.1)	0.00	3,831.3 (10.0)	3,336.7 (9.9)	0.004
Admission to university hospital, n (%)	2,795 (9.2)	1,192 (14.2)	-0.15	3,984.0 (10.4)	3,803.7 (11.3)	-0.03
**Burden of comorbidities**						
Elixhauser comorbidity score, mean (SD)	1.7 (1.0)	2.0 (1.1)	-0.23	1.8 (1.0)	1.8 (1.0)	-0.04
Hospital frailty risk score, n (%)			0.05			<0.001
<5	30,083 (99.3)	8,324 (98.8)		37,999.2 (99.1)	33,467.0 (99.1)	
5-15	212 (0.7)	95 (1.1)		321.1 (0.8)	287.9 (0.9)	
>15	6 (0.0)	3 (0.0)		11.8 (0.0)	5.1 (0.0)	
**Comorbidities, n (%)**						
Obesity classification WHO			0.11			-0.008
Obesity class I (BMI 30.0 - 34.9 kg/m2)	633 (2.1)	118 (1.4)		683.6 (1.8)	454.6 (1.3)	
Obesity class II (BMI 35.0 - 39.9 kg/m2)	11,226 (37.0)	2,780 (33.0)		13,875.5 (36.2)	12,063.3 (35.7)	

© 2025 Wildisen et al. *JAMA Surgery*.

Obesity class III (BMI ≥ 40.0 kg/m2)	17,924 (59.2)	5,332 (63.3)		23,082.1 (60.2)	20,658.1 (61.2)	
Unknown obesity class	518 (1.7)	192 (2.3)		691.0 (1.8)	584.1 (1.7)	
Type 1 diabetes	85 (0.3)	32 (0.4)	-0.02	117.2 (0.3)	116.5 (0.3)	-0.01
Type 2 diabetes	3,994 (13.2)	1,321 (15.7)	-0.07	5,309.9 (13.9)	4,848.6 (14.4)	-0.007
Chronic kidney disease	207 (0.7)	160 (1.9)	-0.11	362.7 (0.9)	353.2 (1.0)	-0.009
Hypertension	8,654 (28.6)	3,052 (36.2)	-0.16	11,682.5 (30.5)	10,860.8 (32.2)	-0.04
Congestive heart failure	85 (0.3)	52 (0.6)	-0.05	140.2 (0.4)	140.8 (0.4)	-0.007
Coronary heart disease	585 (1.9)	284 (3.4)	-0.09	873.4 (2.3)	851.4 (2.5)	-0.01
Atrial fibrillation	237 (0.8)	182 (2.2)	-0.11	419.8 (1.1)	412.2 (1.2)	-0.01
Cerebrovascular disease	21 (0.1)	10 (0.1)	-0.02	30.6 (0.1)	28.7 (0.1)	0.002
Peripheral artery disease	38 (0.1)	24 (0.3)	-0.04	62.0 (0.2)	59.5 (0.2)	0.003
COPD	377 (1.2)	190 (2.3)	-0.08	561.6 (1.5)	535.7 (1.6)	-0.009
OSAS	5,137 (17.0)	1,961 (23.3)	-0.16	7,098.1 (18.5)	6,795.4 (20.1)	-0.04
Dyslipidemia	3,736 (12.3)	1,262 (15.0)	-0.08	4,978.8 (13.0)	4,531.5 (13.4)	-0.01
Hepatopathy	1,189 (3.9)	610 (7.2)	-0.14	1,788.6 (4.7)	1,746.3 (5.2)	-0.02
MASLD	1,174 (3.9)	596 (7.1)	-0.14	1,761.2 (4.6)	1,718.9 (5.1)	-0.02
Osteoporosis	52 (0.2)	20 (0.2)	-0.01	72.3 (0.2)	67.1 (0.2)	0.002
Gastroesophageal reflux disease	6,072 (20.0)	1,224 (14.5)	0.15	7,140.7 (18.6)	5,659.6 (16.8)	0.05
Peptic ulcer disease	62 (0.2)	17 (0.2)	0.00	77.4 (0.2)	68.5 (0.2)	<0.001
Solid cancer	33 (0.1)	14 (0.2)	-0.02	45.4 (0.1)	43.6 (0.1)	-0.003
**Psychiatric disorders, n (%)**						
Psychiatric disorders overall	3,329 (11.0)	1,051 (12.5)	-0.05	4,348.9 (11.3)	3,838.5 (11.4)	0.001
Depression	2,242 (7.4)	688 (8.2)	-0.03	2,904.1 (7.6)	2,600.8 (7.7)	0.004

© 2025 Wildisen et al. *JAMA Surgery*.

Anxiety and obsessive-compulsive disorders	383 (1.3)	119 (1.4)	-0.01	498.2 (1.3)	440.0 (1.3)	<0.001
Substance abuse	287 (0.9)	95 (1.1)	-0.02	381.0 (1.0)	305.0 (0.9)	0.008
Eating disorders	144 (0.5)	21 (0.2)	0.04	161.1 (0.4)	73.5 (0.2)	0.04
**Type of surgery, n (%)**						
Proximal gastric bypass	17,993 (59.4)	n.a.	n.a.	23,582.2 (61.5)	n.a.	n.a.
Roux-en-Y gastric bypass	3,795 (12.5)	n.a.	n.a.	5,030.1 (13.1)	n.a.	n.a.
Distal gastric bypass	731 (2.4)	n.a.	n.a.	954.1 (2.5)	n.a.	n.a.
Omega-loop gastric bypass	289 (1.0)	n.a.	n.a.	380.5 (1.0)	n.a.	n.a.
Not otherwise specified gastric bypass	7,500 (24.8)	n.a.	n.a.	8,394.6 (21.9)	n.a.	n.a.

Data are presented as mean (±SD) for continuous measures, and n (%) for categorical measures.
Abbr.: IPW: inverse probability weighting; SD: Standard deviation; SMD: Standardized mean difference; n: Number; WHO: World Health Organization; BMI: Body-mass index (calculated as weight in kilograms divided by height in meters squared); COPD: Chronic obstructive pulmonary disease; OSAS: Obstructive sleep apnea; MASLD: Metabolic dysfunction-associated steatotic liver disease; n.a.: Not applicable.
[a]Pseudo-population estimates after trimming of 2% of most extreme weights (n = 779)

© 2025 Wildisen et al. *JAMA Surgery*.

eTable 12. Primary and Secondary Outcomes Without Conversion Surgery Cohort and After IPW

	After IPW							
	Gastric bypass			Sleeve gastrectomy				
	**N=29,913**	**N=38,332.2[a]**		**N=8,031**	**N=33,760.1[a]**			
**Outcome**	N (%)	N (%)	IR/1,000 PY	N (%)	N (%)	IR/1,000 PY	HR (95% CI)	P
Primary outcome								
MACE	564 (1.9)	759.8 (2.0)	3.88	234 (2.9)	819.9 (2.4)	5.17	0.72 (0.61 to 0.85)	<0.001
Secondary outcomes								
Myocardial infarction	125 (0.4)	169.5 (0.4)	0.86	61 (0.8)	229.2 (0.7)	1.44	0.56 (0.40 to 0.79)	<0.001
Ischemic stroke	104 (0.3)	139.8 (0.4)	0.71	48 (0.6)	163.0 (0.5)	1.02	0.68 (0.47 to 0.97)	0.03
Hospitalization for heart failure	69 (0.2)	101.9 (0.3)	0.52	34 (0.4)	101.3 (0.3)	0.64	0.78 (0.49 to 1.24)	0.29
All-cause mortality	328 (1.1)	440.1 (1.1)	2.24	118 (1.5)	403.2 (1.2)	2.52	0.85 (0.68 to 1.07)	0.17
Revision surgery[b]	2729 (9.1)	3,411.5 (8.9)	18.46	54 (0.7)	246.8 (0.7)	1.55	12.04 (9.05 to 16.02)	<0.001
GERD and peptic ulcer disease	2238 (7.5)	2,848.9 (7.4)	15.27	500 (6.2)	2,081.5 (6.2)	13.59	1.16 (1.04 to 1.28)	0.006
Hospitalization for dumping syndrome	244 (0.8)	298.6 (0.8)	1.52	14 (0.2)	58.3 (0.2)	0.37	4.18 (2.38 to 7.34)	<0.001
Psychiatric disorders and suicide attempt needing hospitalization	1806 (6.0)	2,303.3 (6.0)	12.20	386 (4.8)	1,598.8 (4.7)	10.34	1.20 (1.07 to 1.34)	0.002

Abbr.: IPW: inverse-probability weighting; N: number; IR: incidence rate; PY: person-years; CI: confidence interval; HR: hazard ratio; P: P-value; MACE: major adverse cardiac event; GERD: gastroesophageal reflux disease

[a]Pseudo-population estimates after IPW and 2% trimming of most extreme weights

[b]Revision meaning any abdominal operation potentially related to the index bariatric procedure but not directly affecting bariatric physiology, including Pyloroplasty, revision gastroenteric anastomosis, Petersen's space revision, implantation or change of a self-expanding endoprosthesis, surgery for hiatal hernia (abdominal/thoracic), and intraoperative manipulation of the stomach.

Cox regression model comparing gastric bypass vs. sleeve gastrectomy after IPW, adjusted for age, sex (female vs. male), nationality (Swiss vs. other nationality), residence before hospital admission (home, nursing home, psychiatric clinic, rehabilitation, penial institution, not known), year of the index-admission, length of hospital stay, hospital site (university hospital vs. other hospitals), coronary heart disease (CHD), heart failure (HF), atrial fibrillation (AFib), cerebrovascular disease, peripheral arterial vascular disease (PAVK), cancer, chronic obstructive pulmonary disease (COPD), obstructive sleep apnea syndrome (OSAS), chronic kidney disease (CKD), obesity, type 1 or 2 diabetes, arterial hypertension, dyslipidemia, hepatopathy, osteoporosis, psychiatric diseases, gastric banding, gastroesophageal reflux disease, peptic ulcer disease, metabolic associated fatty liver disease, Elixhauser comorbidity index and frailty scores.

© 2025 Wildisen et al. *JAMA Surgery*.

eFigure 1. Flowchart

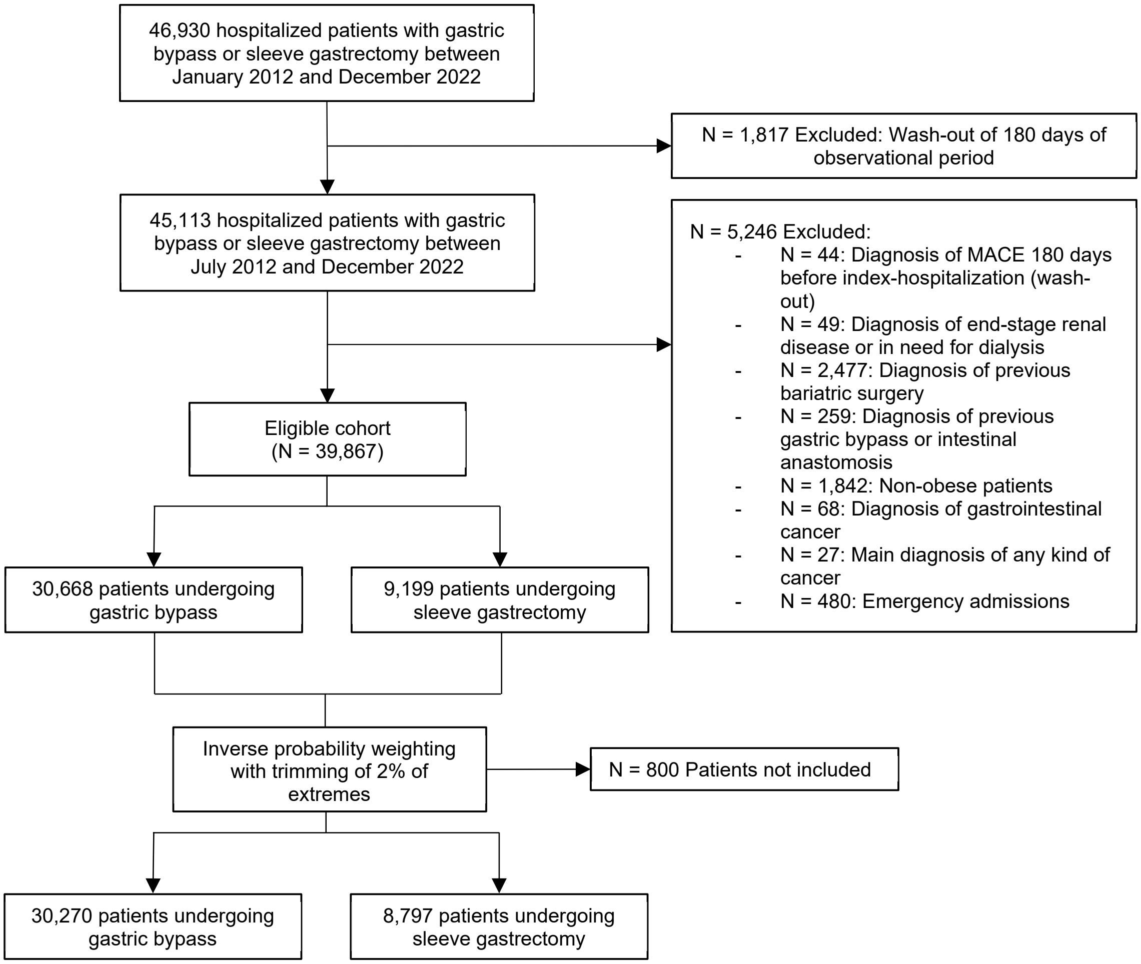

eFigure 2. Density of Propensity-Scores Before Matching, According to Surgical Procedure

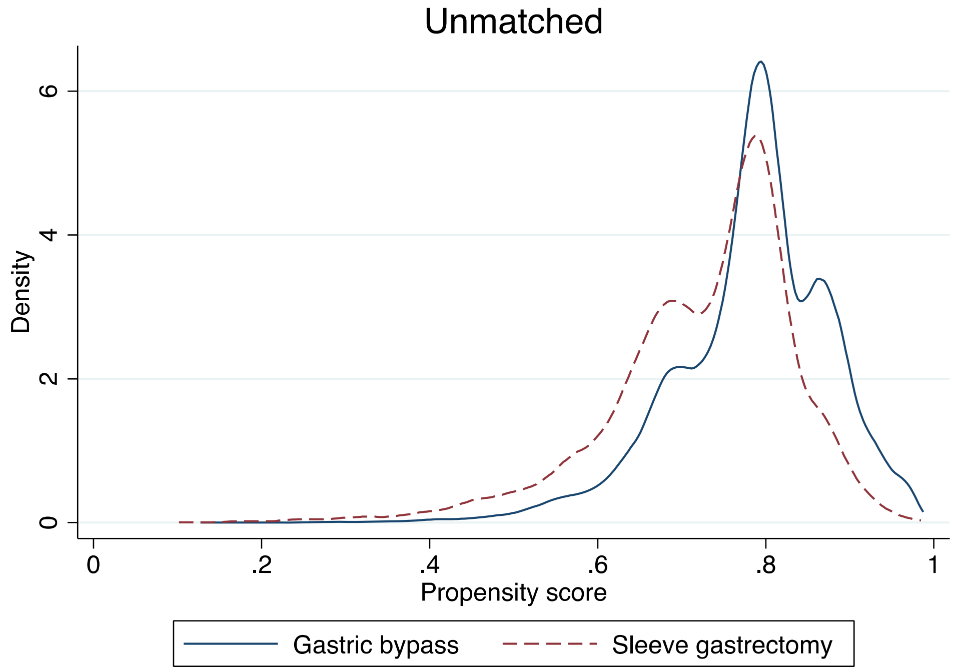

© 2025 Wildisen et al. *JAMA Surgery*.

eFigure 3. Density of Propensity-Scores After Matching, According to Surgical Procedure

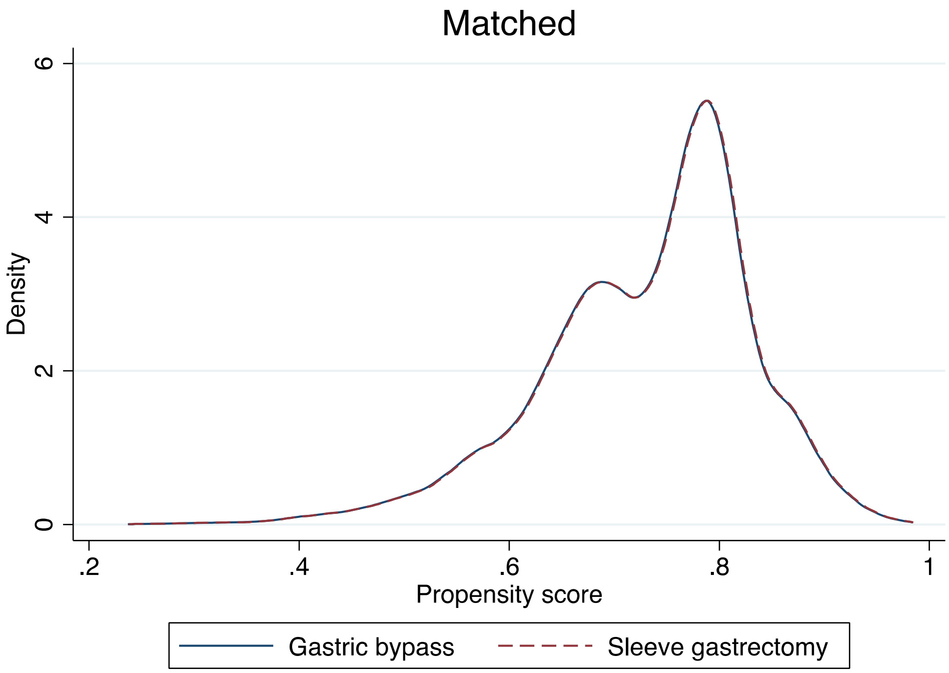

© 2025 Wildisen et al. *JAMA Surgery*.
