# Supplementary material for: Major Adverse Cardiac Events After Gastric Bypass vs Sleeve Gastrectomy
Source: JAMA Surg. 2025 May 7;160(6):690–700. doi: 10.1001/jamasurg.2025.1065 (PMC12060020; doi:10.1001/jamasurg.2025.1065)
Supplement: Supplement 2. — Data Sharing Statement [file jamasurg-e251065-s002.pdf]

Data Sharing Statement

Wildisen. Cardiac Events After Gastric Bypass vs Sleeve Gastrectomy Bariatric Surgery. *JAMA Surg*. Published May 07, 2025. doi:10.1001/jamasurg.2025.1065

Data
Data available: No

Additional Information
Explanation for why data not available: Explanation for why data not available: The data that support the findings of this study are available upon request from the Swiss Federal Statistical Office (Neuchâtel, Switzerland). Restrictions apply to the availability of these data, which were used under license for this study. Data are available as part of the data on “Medizinische Statistik der Krankenhäuser” with the permission of the Swiss Federal Statistical Office, Section Health Services and Population Health.
